# *You talkin’ to me?* Communicative talker gaze activates left-lateralized superior temporal cortex during perception of degraded speech

**DOI:** 10.1016/j.neuropsychologia.2017.04.013

**Published:** 2017-06

**Authors:** Carolyn McGettigan, Kyle Jasmin, Frank Eisner, Zarinah K. Agnew, Oliver J. Josephs, Andrew J. Calder, Rosemary Jessop, Rebecca P. Lawson, Mona Spielmann, Sophie K. Scott

**Affiliations:** a Department of Psychology, Royal Holloway University of London, Egham Hill, Egham TW20 0EX, UK; b Institute of Cognitive Neuroscience, University College London, 17 Queen Square, London WC1N 3AR, UK; c Donders Institute, Radboud University, Montessorilaan 3, 6525 HR Nijmegen, Netherlands; d Department of Otolaryngology, University of California, San Francisco, 513 Parnassus Avenue, San Francisco, CA, USA; e Wellcome Trust Centre for Neuroimaging, Institute of Neurology, University College London, 12 Queen Square, London WC1N 3BG, UK; f MRC Cognition and Brain Sciences Unit, 15 Chaucer Road, Cambridge CB2 7EF, UK

**Keywords:** Gaze, FMRI, Speech comprehension, Laterality

## Abstract

Neuroimaging studies of speech perception have consistently indicated a left-hemisphere dominance in the temporal lobes’ responses to intelligible auditory speech signals (McGettigan and Scott, 2012). However, there are important communicative cues that cannot be extracted from auditory signals alone, including the direction of the talker's gaze. Previous work has implicated the superior temporal cortices in processing gaze direction, with evidence for predominantly right-lateralized responses (Carlin & Calder, 2013). The aim of the current study was to investigate whether the lateralization of responses to talker gaze differs in an auditory communicative context. Participants in a functional MRI experiment watched and listened to videos of spoken sentences in which the auditory intelligibility and talker gaze direction were manipulated factorially. We observed a left-dominant temporal lobe sensitivity to the talker's gaze direction, in which the left anterior superior temporal sulcus/gyrus and temporal pole showed an enhanced response to direct gaze – further investigation revealed that this pattern of lateralization was modulated by auditory intelligibility. Our results suggest flexibility in the distribution of neural responses to social cues in the face within the context of a challenging speech perception task.

## Highlights

• Talker gaze is an important social cue during speech comprehension.
• Neural responses to gaze were measured during perception of degraded sentences.
• Gaze direction modulated activation in left-lateralized superior temporal cortex.
• Left lateralization became stronger when speech was less intelligible.
• Results suggest task-dependent flexibility in cortical responses to gaze.

## 1 Introduction

Spoken communication can only be described in part by reference to the exchange of linguistic messages. Natural conversation often occurs face-to-face, where interlocutors display facial expressions, gestures and non-verbal vocalizations (such as laughter) in order to enhance understanding, and to signal social cues such as mood, affiliation and intent. However, to date, relatively little is known about how the brain processes social and linguistic cues within the same communicative context.

Neuroimaging studies of auditory speech intelligibility in the healthy adult brain have attempted to isolate the neural responses to intelligible (or partially intelligible) speech signals by comparison with acoustically complex, unintelligible control conditions (Eisner et al., 2010, Evans et al., 2014, Narain et al., 2003, Scott et al., 2000) and by using parametric modulations of speech intelligibility, for example by varying the number of channels in noise-vocoded speech (Davis and Johnsrude, 2003a, Davis and Johnsrude, 2003b; McGettigan et al., 2012a, McGettigan et al., 2012b; Obleser et al., 2007; Scott et al., 2006). This work has identified that the process of extracting an intelligible message from an auditory speech signal engages an anterior-going pathway in the superior temporal lobes (Evans et al., 2014, Scott et al., 2000) as well as responses in the inferior frontal gyrus (IFG), anterior insula and premotor cortex (Adank, 2012a; Davis and Johnsrude, 2003a, Davis and Johnsrude, 2003b; Eisner et al., 2010; Hervais-Adelman et al., 2012; McGettigan et al., 2012b). Speech comprehension can also be manipulated experimentally through alternate methods, such as the comparison of words with pseudowords, and the use of semantic and syntactic violations, revealing similar loci (see Adank, 2012b). Although some authors argue that the perceptual processing of speech is bilateral in the temporal lobes (Hickok and Poeppel, 2007, Okada et al., 2010) our work has strongly suggested a left hemisphere dominance for intelligible speech perception (including perception of sentences, words, syllables, phonemes, syntactic and semantic information; see McGettigan and Scott, 2012), with a complementary right-hemisphere dominance for the processing of melodic aspects of spoken signals and the perception of vocal identities (Scott et al., 2000, Kyong et al., 2014, McGettigan and Scott, 2012).

Previous investigations of audiovisual speech intelligibility have shown that the presence of dynamic facial cues improves speech report accuracy under difficult listening conditions (McGettigan et al., 2012b, Ross et al., 2007, Sumby and Pollack, 1954). Neuroimaging studies of audiovisual speech perception have implicated sites including the posterior superior temporal sulcus (STS), inferior parietal cortex, motor cortex and subcortical structures such as the caudate nucleus (Bernstein et al., 2008, Calvert et al., 1997, Calvert et al., 2001, McGettigan et al., 2012b, Skipper et al., 2005, Stevenson and James, 2009). However, beyond the basic cues to speech intelligibility from the movements of the lips and facial muscles, a talking face brings other information to a communicative interaction, including cues to mood and intentionality - salient amongst these cues is the gaze of the talker. Senju and Johnson (2009) consider the behavioural and neural effects of experiencing eye contact with another person. The authors identify a set of key brain regions that are regularly implicated in studies of gaze perception from faces, including the fusiform gyrus, anterior and posterior portions of the STS, medial prefrontal and orbitofrontal cortices, and the amygdala. They describe how perceived eye contact from another can increase autonomic arousal and modulate activation within the “social brain” (medial prefrontal cortex (mPFC), temporal poles and the temporo-parietal junction (TPJ)), thus signaling communicative intent to this system. However, they also note inconsistencies in the neuroimaging literature on gaze perception, where some brain regions are only implicated across some studies, while other regions show contradictory responses from one study to the next (e.g. mPFC showing a preferential response to direct eye contact in one study, but to averted gaze in another). To make sense of these inconsistencies, Senju and Johnson propose their “fast-track modulator” model of eye gaze, in which they suggest that the fundamental mechanism for eye gaze detection is subcortical in its origin, and that the involvement of higher-order cortical centres is strongly dependent on task demands.

The STS has been a key region implicated in both the perceptual processing of both speech (Binder et al., 2000; Davis and Johnsrude, 2003a, Davis and Johnsrude, 2003b; Eisner et al., 2010; Evans et al., 2014; Liebenthal et al., 2005; Scott et al., 2000) and eye gaze direction (Calder et al., 2006, Calder et al., 2002, Carlin et al., 2011, Hoffman and Haxby, 2000, Hooker et al., 2003, Pelphrey et al., 2003, Pelphrey et al., 2004, Puce et al., 1998). Senju and Johnson (2009) describe a difficulty in resolving the relative roles of posterior and anterior sites on the sulcus in terms of the response to eye contact and the mechanisms for discriminating gaze direction, where they suggest that directed attention to the eyes may be required to activate the anterior STS while posterior sites may require dynamic visual cues and/or conscious recognition of communicative intent from the viewed person. Calder and colleagues carried out a series of studies in which they attempted to better resolve mechanisms for gaze processing along the STS (Calder et al., 2006, Calder et al., 2002, Carlin and Calder, 2013, Carlin et al., 2011). In line with evidence from single-cell recordings in monkeys (e.g. Perrett et al., 1992; Perrett et al., 1985), Carlin et al. (2011) characterized an anterior-going processing hierarchy in the STS, where posterior sites are sensitive to both gaze and head direction while the anterior STS shows head-direction-invariant responses to gaze. In this way, it is suggested that the anterior STS is more responsive to the social significance of gaze than to the specific configurations of the visual cues that signal it. Here, there are strong parallels with the speech perception literature, in which there is a long-standing debate over whether the crucial mapping of sound to linguistic representations takes place primarily in posterior or anterior STS (Evans et al., 2014, Okada et al., 2010, Scott et al., 2000). There is an argument for a speech processing hierarchy in humans that is homologous to the ventral “what” pathway for auditory object recognition in the temporal lobe of non-human primates (Rauschecker and Scott, 2009, Scott and Johnsrude, 2003c). The anterior STS forms the highest point in this hierarchy, being responsive to intelligible speech signals regardless of their specific acoustic properties (e.g. whether they are undistorted or spectrally degraded; Scott et al., 2000). However, the main distinction between the findings with gaze and speech perception is one of hemispheric lateralization, where responses to speech tend to be left dominant in the superior temporal lobes (McGettigan and Scott, 2012), while sensitivity to gaze direction is more consistently *right-lateralized* (Carlin and Calder, 2013).

It is tautological to think of “social speech processing”, given that the vast majority of spoken language interactions take place in social settings. Nonetheless, the neurobiological literature has relatively little to say about social context for spoken communication in terms of how the processing of auditory information might interact with other social cues in speech perception (Scott et al., 2009). Above, we note the potential commonalities of anterior-going temporal lobe hierarchies for the processing of auditory speech and eye gaze direction. The STS has been repeatedly implicated in the processing of socially-relevant signals, including emotional prosody, facial expressions, vocal identity, gesture and biological motion (Allison et al., 2000, Belin et al., 2000, Grandjean et al., 2005, Grezes et al., 2003). In an attempt to unify this response profile in terms of its underlying computations, Redcay (2008) has proposed that the primary function of the STS is to parse and interpret the communicative significance of incoming streams of audio, visual and audiovisual information unfolding over time. Recent work using vocal signals supports this suggestion of the STS as a locus for social perception, finding that communicative speech and emotional vocalizations generated greater responses in the STS than non-communicative sneezes and coughs (Shultz et al., 2012). Similarly, the right posterior STS has been found to be specifically involved in the planning and perception of communicative (vs. non-communicative) actions in a two-player computer game (Noordzij et al., 2010, Stolk et al., 2013).

Given the apparent parallels between gaze and speech perception pathways, yet a strong difference in the reported lateralization of these processes, an unanswered question is whether and how the lateralization of one or both processes might be affected by the task context. Behaviourally, there is evidence that heard speech can influence the perception of gaze in a simultaneously presented face – participants were more likely to label gaze direction in a static face as direct when an accompanying voice called the participant's name (vs. a control name; Stoyanova et al., 2010). Kampe and colleagues (Kampe et al., 2003) presented participants with visual and auditory stimuli in separate trials of an event-related fMRI experiment. In the visual condition, they manipulated the gaze of static faces to be direct or averted with respect to the participant, while in the auditory condition a heard voice either demanded the attention of the participant by name (e.g. “Hey John!”) or addressed another person. Within each modality, the authors found modulation of key sites in the social brain by conditions with greater communicative intent (i.e. direct gaze and use of the participant's name) - the paracingulate cortex and the left temporal pole were implicated for both modalities. This is an important indication that there is sensitivity to gaze, and its social significance, in the left as well as the right temporal cortex. Using a region of interest analysis, Carlin et al. (2011) also reported head-view-invariant responses to gaze direction in the left anterior STS.

In Senju and Johnson's (2009) proposed “fast-track modulator” model of the eye contact effect, a subcortical stream forms the first path for detection of eye contact and projects to several sites in the social brain. These, in interaction with dorsolateral prefrontal responses to task demands and social context, then influence the further processing of gaze cues in the cortex. Although this model is focused on the specific percept yielded when another's eyes make direct contact with the gaze of the perceiver, its broad implication is that there is a dynamic network, or set of networks, underpinning the extraction of gaze cues in terms of their social and communicative significance. The aim of the current study was explore the neural responses to talker gaze direction in the context of a speech intelligibility task. We had a particular interest in how the context of a challenging speech task, in which the left hemisphere dominates, would interact with a manipulation that has, in the existing literature, predominately engaged the right temporal lobe. Specifically, we predicted that if participants are primarily engaged in trying to understand what a talker is saying, this could lead to a stronger left-lateralization (or weaker right-lateralization) of superior temporal gaze responses, reflecting dynamic alignment of cortical processes according to the task at hand.

## 2 Methods

### 2.1 Materials

#### 2.1.1 Stimuli

The stimuli were 240 English sentences chosen from the BKB (Bamford-Kowal-Bench) list, each featuring three key words (e.g. ‘The CLOWN had a FUNNY FACE’; Bench et al., 1979). The sentences were spoken by a female speaker of Standard Southern British English. The talker, seated, delivered each sentence with three different gaze directions: Direct Gaze (looking directly into the camera lens), Averted Gaze (with gaze held on a single fixed point marked to the right of the camera), Downward Gaze (with head upright, but eyes cast downward toward the talker's lap).

The videos were filmed in a soundproof room, with the talker's face set against a blue background and illuminated with a key and a fill light. The talker's head was fully visible within the frame. Video recordings were made to a Canon XL-1 DV camcorder, with a Bruel & Kjaer type 4165 microphone.

In Final Cut Pro (Apple, Cupertino, CA), the orientation of the raw video was slightly adjusted so that the head was straight and centered. The onset and offset of each sentence was marked (using neutral mouth starting and final positions), and the sentences were exported as individual clips in QuickTime (.mov) format. The mean duration of these raw video clips was 2.99 s (s.d. 0.39 s, range 1.84–3.96 s). In Praat (Boersma and Weenink, 2008), the audio tracks were normalized for peak amplitude, and a cosine ramp was applied at on- and offset. The files were noise-vocoded using a custom-built script in Matlab (The Mathworks, Natick, MA) by passing the speech waveform through a bank of either 2 or 3 analysis filters (upper cutoff 11025 Hz). The filter bandwidths were set to represent equal distances along the basilar membrane (according to the Greenwood, 1990 equation relating filter position to best frequency). The amplitude envelope was extracted at the output of each analysis filter via half-wave rectification and low-pass filtering at 400 Hz. The envelopes were each then multiplied with a band-limited white noise carrier, filtered and summed together. The re-summed stimulus was low-pass filtered at 11025 Hz. Finally, the audio versions of each sentence (with 2 or 3 channels) were combined with their respective video using a shell script.

**Fig. 1:**
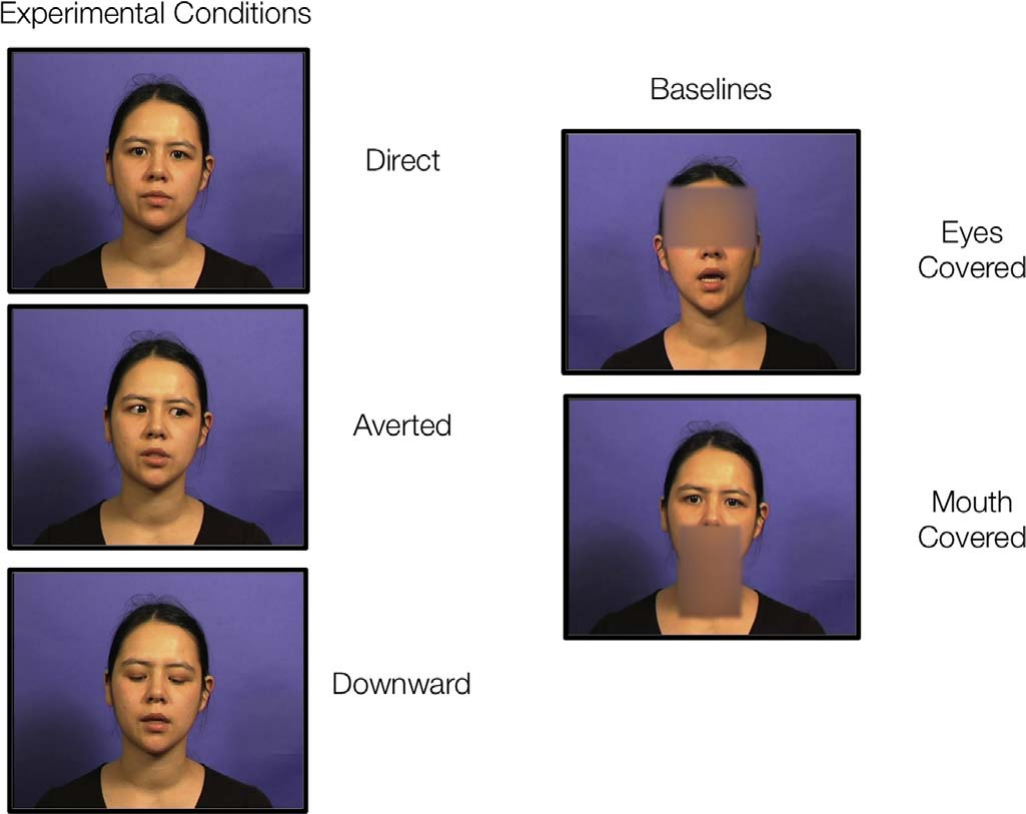
Example frames from the visual conditions used in the study. Please note that the Mouth Covered baseline was used in the behavioural pilot experiment only.

In order to balance the laterality of gaze in the Averted condition, Final Cut Pro was used to horizontally flip half of the sentences in all conditions (Averted, Direct, Downward). Finally, two baseline conditions were created. For each item, a rectangular patch (150x100 pixels) was blurred (with radius 150 pixels) using the Joe's Soft Shapes plugin in Final Cut Pro (www.joesfilters.com). The rectangle was positioned horizontally to cover the eyes (Eyes Covered baseline) or vertically to obscure motion cues from the mouth and throat (Mouth Covered baseline). Fig. 1 shows an example frame from each of the five visual conditions.

All videos were saved at 720×576 pixels in size, at 25 frames per second, with 16-bit audio at sample rate 22050 Hz.

### 2.2 Behavioural pilot experiment

Sixteen native speakers of British English (aged 18–40 years old) took part in a behavioural sentence report experiment adopting a 5×2 factorial design, with the factors Visual Condition (Averted Gaze, Direct Gaze, Downward Gaze, Eyes Covered, Mouth Covered) and Auditory Clarity (2 vs. 3 noise-vocoded channels, where the latter should be of higher intelligibility due to the presence of greater spectral detail). The experiment was conducted with approval from the UCL Research Ethics Committee.

In total, there were 240 trials falling into three blocks of 80 trials. Within each block, there were 10 trials from each of the main experimental conditions (3 gaze directions×2 levels of auditory clarity), and 5 from each of the mouth and eyes baselines. These were presented in a pseudorandomized order, in which miniblocks of 16 trials featured 2 examples from each of the main experimental conditions and one from each of the baseline conditions (Mouth Covered and Eyes Covered). The stimuli were presented onscreen and over headphones (Sennheiser HD-210, Sennheiser electronic GmbH & Co. KG, Wedermark, Germany) from a MacBook Pro laptop (15″ screen, resolution 1440×900 pixels; Apple Inc., Cupertino, CA) running Matlab (Version R2009a; The Mathworks, Natick, MA) with the Psychophysics Toolbox extension (Brainard, 1997). After each sentence was played, the participant typed what they understood from the sentence, and pressed Enter to advance to the next trial. There was no time limit on responses, and participants were given the opportunity to take breaks between blocks. The participants’ responses across all ten conditions were individually scored in terms of the proportion of Key Words that were correctly reported from each sentence.

**Fig. 2:**
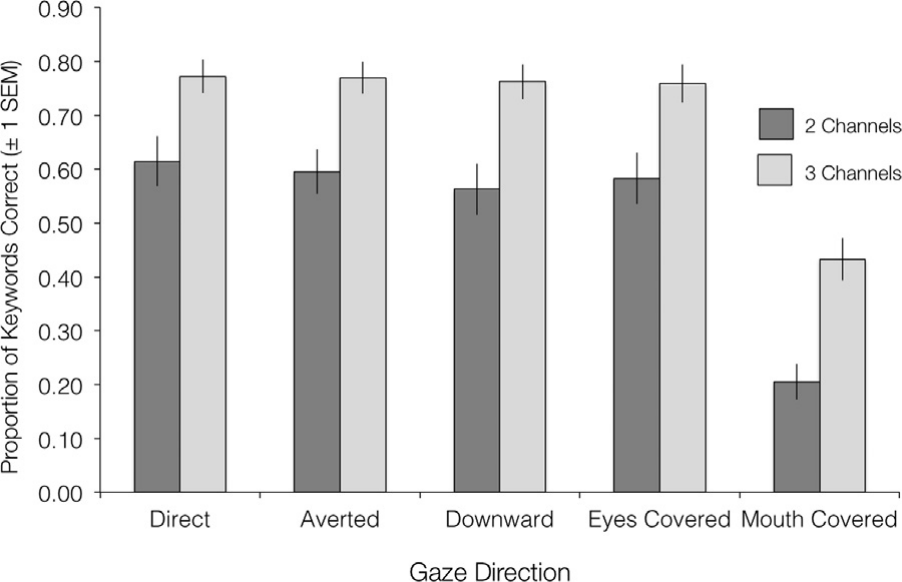
Plot of mean group accuracy (±1 S.E.M.) on a test of sentence report, across the factors Visual Condition and Auditory Clarity.

Fig. 2 shows the mean sentence report performance across all conditions). A 5×2 repeated-measures ANOVA revealed a significant main effect of Auditory Clarity (F(1,15)=124.54, p<0.001, partial eta sq.=0.893) and a significant main effect of Visual Condition (F(4,60) =105.20, p<0.001, partial eta sq.=0.875). Bonferroni-corrected pairwise comparisons revealed that performance on the Mouth Covered condition was significantly worse than on all other visual conditions (all ps <0.001). The interaction of Auditory Clarity and Visual Condition was non-significant (F(4,60) =1.34, p=0.082, partial eta sq.=0.082). A 3×2 repeated-measures ANOVA on the six experimental conditions for the MRI experiment with factors Gaze Direction (Direct, Averted, Downward) and Auditory Clarity (Channels: 2 and 3) revealed a significant main effect of Auditory Clarity (F(1,15) =104.31, p<0.001, partial eta sq.=0.874). There was a non-significant effect of Gaze Direction (F(2,30)=1.37, p=0.270, partial eta sq.=0.084) and a non-significant interaction of the two factors (F(2,30)=1.63, p=0.214, partial eta sq.=0.098).

Thus, in line with the existing literature, the behavioural experiment showed that viewing mouth movements can enhance intelligibility of speech in the presence of a degraded auditory signal. However, the direction of gaze had no significant effect on speech comprehension performance. It might have been expected that direct gaze, being socially salient, might somehow orient attention and thus yield improved sentence report scores – the absence of this effect means, however, that any observed effect of gaze direction in the BOLD signal in STS could not simply be ascribed to differences in linguistic comprehension as a consequence of modulations in visual attention.

### 2.3 Functional magnetic resonance imaging (fMRI)

In the interests of maximizing power in the functional imaging experiment, the Mouth Covered baseline condition used in the behavioural pilot was not included. Thus, participants were presented with a 2×3 array of audiovisual sentence conditions with two levels of auditory clarity (2 and 3 channels) and three gaze directions (Direct, Averted, Downward), plus the Eyes Covered baseline (at both levels of auditory clarity) and a rest baseline.

### 2.4 Participants

Participants in the study were 18 adults (7 male; mean age 23 years,^1^ range 20–27 years) who spoke English as their first language. All were right-handed, with healthy hearing and no history of neurological incidents, nor any problems with speech or language (self-reported). The study was approved by the UCL Research Ethics Committee.

### 2.5 Procedure

The experiment followed a 2×4 design with factors Gaze Direction (Averted, Downward, Direct, None) and Channels (2, 3). A sparse-sampling routine (Edmister et al., 1999, Hall et al., 1999) was employed, in which the audiovisual stimuli were presented in the quiet period between scans. In total, there were 30 trials from each of these eight conditions, organized into miniblocks of 16 randomized trials containing 2 examples from each condition. After each miniblock of 16 trials, a seventeenth “catch” trial contained a 1-back vigilance task, in which the participant was probed with an onscreen text keyword and asked to indicate (via keypress on the in-scanner button box) whether this word was contained in the most recently played sentence. The selected keyword came from the preceding trial on 50% of occasions, and on the other 50% was selected randomly from one of the BKB sentences used in the experiment. The catch trial responses were not analysed due to the 50% chance rate and small number of trials (15). In addition to the video and catch trials, there were three miniblocks of silent baseline trials, each lasting for 5 trials, which occurred around the midpoint of each functional run. During these trials, the participant saw the words “Mini Break…” written on the screen.

Functional imaging data were acquired on a Siemens Avanto 1.5-Tesla MRI scanner (Siemens AG, Erlangen, Germany) with a 32-channel head coil. Audio-visual presentation of sentences took place in three runs of 95 whole-brain volumes using a dual-echo echo-planar imaging sequence (TR=9 s, TA=3.7 s, TE =24; 58 ms, 3 mm×3 mm×3 mm in-plane resolution, 40 slices with 25 degree tilt transverse to coronal, ascending sequential acquisition).

Each video trial began with a 3-second presentation of a fixation cross against a black background. This was positioned roughly at the midpoint between the talker's eyes in the upcoming videos and was presented simultaneously with the onset of the whole-brain volume. Video onsets were timed such that the mid-point of each video occurred 5 s before the mid-point of the following whole-brain EPI volume acquisition. By using the variability in durations as a “natural jitter”, this resulted in onsets varying across a window of 1.06 s (i.e. the difference in the onsets of the longest and shortest videos: (3.96 – 1.84)/2).

Stimuli were presented using MATLAB with the Psychophysics Toolbox extension, on a MacBook Pro laptop computer (15″ screen, resolution 1440×900 pixels; Apple Inc., Cupertino, CA). The audio channel was routed through a Sony HD-510 amplifier (Sony Europe Limited, Weybridge, UK) to electrodynamic MR-compatible headphones worn by the participant (Sensimetrics Corporation, Malden, MA). Videos were presented at a resolution of 1024×768 pixels from an EPSON EH-TW5900 projector (Seiko Epson Corp., Nagano, Japan) to a custom-built screen at the back of the scanner bore, which was viewed using a mirror placed on the head coil. Responses to the catch trials were collected via an MR-compatible optical LUMItouch response keypad (Photon Control, Inc., Burnaby, Canada). After the functional run, a high-resolution T1-weighted anatomical image was acquired (HIRes MP-RAGE, 160 sagittal slices, voxel size=1 mm3).

The total time in the scanner was around 1 h. As part of the experiment, participant pupil size and gaze direction were measured during the fMRI data acquisition using an Eyelink 1000 MR-compatible eye tracking system (SR Research Ltd., Ottowa, Canada). However, due to technical issues with the labeling of stimulus events, we were unfortunately unable to analyse these data.

### 2.6 Analysis of fMRI data

Analysis of the MRI data was carried out using SPM8 (Wellcome Trust Centre for Neuroimaging, London, UK). The analysis of EPI data used whole-brain volumes collected on the second echo (TE =58 ms) only. Functional images were realigned and unwarped, co-registered with the anatomical image, normalized using parameters obtained from unified segmentation of the anatomical image (involving resampling to isometric voxels of 2×2×2 mm), and smoothed using a Gaussian kernel of 8 mm FWHM.

At the single-subject level, event onsets from all conditions (4 gaze×2 channels, plus catch trials) were modeled using a finite impulse response basis function (length: 1 scan, order: 1) in SPM8, along with six movement parameters of no interest. Contrast images for each condition against the implicit baseline (comprising all silent rest trials) were calculated in the single subject and taken forward to a second-level, random effects 3×2 within-subjects flexible factorial ANOVA model in SPM8, with factors Subject, Gaze Direction (Direct, Downward, Averted) and Auditory Clarity (2,3). Here, we decided not to model the Eyes Covered conditions in the ANOVA because we did not want to conflate a manipulation of eyes present vs. eyes absent with one of gaze direction. From this 3×2 model, F contrast images were calculated for the Main Effect of Gaze ([kron([1 1], orth(diff(eye(3))')'], Main Effect of Auditory Clarity ([−1−1−1 1 1 1]) and the Interaction of Gaze Direction and Auditory Clarity ([kron([1−1], orth(diff(eye(3))')']), as well as T contrasts describing the effect of increasing Auditory Clarity (3 Channels >2 Channels; [−1−1−1 1 1 1]), the response to Direct Gaze (> Downward and Averted; [2−1−1 2−1−1]), Averted Gaze (> Direct and Downward; [−1 2−1−1 2−1]) and Downward Gaze (> Averted and Direct; [−1−1 2−1−1 2]), and the combined response to Direct Gaze and Averted Gaze (> Downward; [1 1−2 1 1−2]). To allow for an exploration of changes in laterality with speech intelligibility (see below), additional one-way within-subjects ANOVAs with the single factor Gaze Direction (Direct, Downward, Averted) were run separately for the two levels of Auditory Clarity (2 Channels, 3 Channels). Finally, to allow for pairwise comparisons of gaze conditions and their interactions with speech intelligiblity, three within-subjects ANOVAs with factors Gaze Direction and Auditory were run for (i) Direct vs. Downward, (ii) Averted vs. Downward and (iii) Direct vs. Averted – the results of these analyses can be found in the Supplemental Material.

All second-level models were calculated at a voxelwise threshold of p<0.005 (uncorrected). A cluster extent correction of 68 voxels (544 mm^3^) was applied for a whole-brain alpha of p<0.001 using a Monte Carlo simulation (with 10 000 iterations) implemented in MATLAB (with smoothness estimate of 13.2 mm; Slotnick et al., 2003).

Second-level peak coordinates were used to extract condition-specific parameter estimates from 4 mm-radius spherical regions of interest (ROIs) built around the peak voxel (using MarsBaR; Brett et al., 2002). The anatomical locations of peak and sub-peak voxels (at least 8 mm apart) were labelled using the SPM Anatomy Toolbox (version 20) (Eickhoff et al., 2005).

### 2.7 Calculating laterality indices

To test the temporal lobe lateralization of activation in the Main effect of Gaze Direction, Main Effect of Auditory Clarity, Positive Effect of Auditory Clarity (2<3) and the preferential response to Direct gaze (> Averted and Downward), we used the LI toolbox in SPM8 (https://www.medizin.uni-tuebingen.de/kinder/en/research/neuroimaging/software/?download=li-toolbox; Wilke and Schmithorst, 2006). For each contrast of interest, the toolbox calculates laterality indices (LI) using the equation: LI=(Σactivationleft –Σactivationright /Σactivationleft +Σactivationright), where Σ refers to the sum of activation either in terms of the total voxel count, or the sum of the voxel values within the statistical map of the contrast. Thus, values, of LI can vary from +1 (completely left lateralized) to −1 (completely right lateralized). According to convention, an absolute LI value greater than 0.2 is taken to indicate a hemispheric dominance (Seghier, 2008). In this paper, “activation” in the LI formula was defined as the total voxel values within each hemisphere in the second-level 3×2 flexible factorial ANOVA F maps of the Main Effect of Gaze Direction and Main Effect of Auditory Clarity, and the T maps of the Positive Effect of Auditory Clarity and preferential response to Direct gaze (over Averted and Downward), restricted in our case to the left and right temporal lobes (defined using an inclusive bilateral anatomical mask of the superior and middle temporal gyri and temporal poles constructed from the AAL regions of interest available in the Marsbar toolbox; Brett et al., 2002). LIs were also calculated for the contrast Direct Gaze >(Averted and Downward) for separate one-way within subject ANOVAs using conditions with 2 channels only, and 3 channels only. To take account of thresholding effects, the toolbox calculates LIs at 20 thresholding intervals from 0 to the maximum value in the F/T map. At each level, the toolbox selects 100 bootstrap samples (5–1000 voxels) from each masked hemisphere, which are paired in all possible combinations (10,000) and used to calculate an equivalent number of LIs. From the final distribution of LIs, the toolbox reports trimmed means (where the top and bottom 25% of values have been discarded), as well as a single weighted mean based on these that is proportionally more affected by LI values from higher statistical thresholds. Here, we report the trimmed and weighted means for each contrast of interest.

## 3 Results

### 3.1 The left temporal lobe is preferentially responsive to increasing intelligibility of degraded speech

**Fig. 3:**
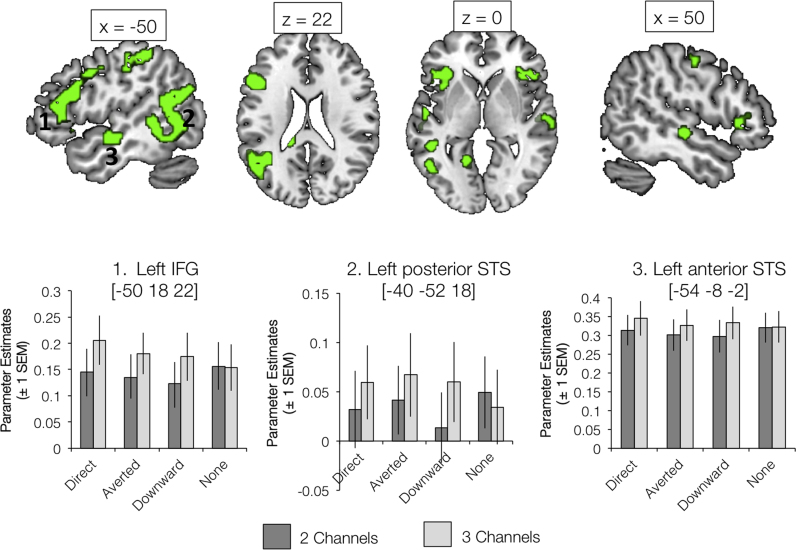
Significant clusters showing a main effect of Auditory Clarity. Activations are shown at a voxel height threshold of p<0.005 and a corrected cluster extent threshold of p<0.001 (Slotnick et al., 2003). Plots show parameter estimates (±1 S.E.M.) taken from 4 mm-radius spherical regions-of-interest built around selected peak voxels (using the MarsBaR toolbox in SPM; Brett et al., 2002). Coordinates are given in Montreal Neurological Institute stereotactic space.

A contrast exploring the main effect of Auditory Clarity revealed significant clusters in regions of superior temporal sulcus (both posterior and anterior) and inferior frontal (extending into premotor) cortex, as found in several previous studies (Davis and Johnsrude, 2003a, Davis and Johnsrude, 2003b; Eisner et al., 2010; McGettigan et al., 2012a, b). (see Fig. 3 and Table 1). These all showed a similar profile, where there were larger responses to sentences with 3 noise-vocoded channels (and hence greater auditory clarity) than to sentences with 2 noise-vocoded channels. The activation in the superior temporal lobes was strongly left lateralized (according to the weighted conventional threshold of >0.2; Seghier et al., 2008. See Table 2).

**Table 1:** Results of the main contrasts exploring the effects of Gaze Direction and Auditory Clarity. All results are reported at a voxel height threshold of p<0.005 (uncorrected), and a corrected cluster threshold of p<0.001 (Slotnick et al., 2003). Coordinates are given in Montreal Neurological Institute (MNI) stereotactic space.

Contrast	No of voxels	Region (s)	Peak Coordinate			F/T	Z
			x	y	z		
Main Effect of Gaze Direction (F-test)	2680	Right/left calcarine gyrus and left cuneus	24	−62	10	16.25	4.74
	69	Right inferior parietal lobule	62	−50	44	14.17	4.42
	186	Right IFG (pars triangularis)	36	28	2	13.95	4.39
	111	Left cerebellum (lobules IV-V)	−12	−50	−12	10.91	3.84
	119	Left STG/STS/temporal pole	−62	−2	−6	10.87	3.84
	118	Left precuneus	−6	−80	46	10.06	3.67
	316	Right superior and inferior parietal lobule; right post-central gyrus	20	−48	58	9.76	3.61
	68	Right postcentral gyrus	60	−4	20	8.93	3.43
	115	Right superior/middle frontal gyrus	26	−2	54	8.79	3.40
	82	Left posterior-medial frontal lobe; left mid-cingulate cortex	−8	10	46	7.99	3.21

Main Effect of Auditory Clarity (F-test)	1031	Left IFG (pars triangularis, orbitalis); left precentral gyrus; left insula	−50	18	22	19.13	3.98
	223	Left superior/middle frontal gyrus	−22	−6	52	18.18	3.88
	136	Left superior frontal gyrus	−10	52	36	17.13	3.77
	1658	Left STS/STG; left middle occipital gyrus	−40	−52	18	17.00	3.76
	166	Right STS/STG	50	−16	−6	16.48	3.70
	227	Bilateral posterior-medial frontal lobe	4	20	50	16.26	3.67
	260	Left mid-cingulate cortex; right posterior-medial frontal lobe	0	0	34	15.79	3.62
	247	Right IFG (pars triangularis); right insula	44	30	6	15.35	3.57
	104	Left lingual gyrus	−14	−56	2	15.33	3.57
	195	Left medial temporal lobe (white matter)	−28	−50	8	12.91	3.27
	68	Left postcentral gyrus	−32	−38	44	12.94	3.21
	113	Left central sulcus/post-central gyrus	−24	−28	52	11.99	3.14
	150	Bilateral cuneus	4	−52	62	11.85	3.12
	73	Right precentral gyrus/central sulcus	56	−14	46	11.65	3.10
	240	Left STG/STS	−54	−8	−2	11.57	3.08

3 channels > 2 channels (T-test)	1530	Left IFG (pars triangularis, orbitalis); left precentral gyrus; left insula	−50	18	22	4.37	4.14
	343	Left superior/middle frontal gyrus	−22	−6	52	4.26	4.05
	353	Left superior frontal gyrus; bilateral superior medial gyrus; right middle frontal gyrus	−10	52	36	4.14	3.94
	3466	Left STS/STG; middle occipital gyrus	−40	−52	18	4.12	3.93
	380	Right STS/STG	50	−16	−6	4.06	3.87
	978	Bilateral posterior-medial frontal lobe; bilateral mid-cingulate cortex	4	20	50	4.03	3.85
	459	Right IFG (pars triangularis); right insula	44	30	6	3.92	3.75
	354	Bilateral lingual gyrus; bilateral calcarine gyrus	−14	−56	2	3.92	3.74
	90	Left putamen	−32	−8	−6	3.61	3.47
	289	Bilateral precuneus	4	−52	62	3.44	3.32
	72	Right putamen	30	−4	0	3.44	3.32
	175	Right precentral gyrus / central sulcus	56	−14	46	3.41	3.30
	518	Left STS/STG	−54	−8	−2	3.40	3.28
	84	Right superior parietal lobule	20	−56	54	2.93	2.85

Direct > Averted & Downward	2781	Bilateral calcarine gyrus; right superior occipital gyrus; left cerebellum (lobules IV-V)	24	−60	10	4.72	4.43
(T-test)	373	Left STG/STS; temporal pole; left Rolandic operculum; left postcentral gyrus	−62	−2	−6	4.56	4.30
	69	Right cerebellum (lobules VIIIa, VI)	38	−46	−38	4.55	4.29
	139	Right IFG (pars triangularis)	34	30	2	4.21	4.00
	268	Right cerebellum (lobules IV-V, VI)	32	−38	−26	4.07	3.88
	357	Left IFG (pars triangularis)	−42	22	0	3.70	3.55
	125	Right insula	44	2	6	3.63	3.49
	68	Left fusiform gyrus	−32	−42	−24	3.57	3.44
	95	Right postcentral gyrus	60	−4	20	3.57	3.43
	94	Right postcentral gyrus	42	−22	36	3.49	3.37
	70	Left superior occipital gyrus; right cuneus	−4	−84	46	3.46	3.34
	83	Right cerebellum (lobules VIIIa, VI)	8	−82	−28	3.30	3.19
	125	Left superior frontal gyrus	−28	60	14	3.22	3.12

Averted > Direct & Downward (T-test)	626	Right superior/inferior parietal lobule; right precuneus	20	−48	58	4.21	4.00
	77	Left inferior parietal lobule	−66	−32	36	3.81	3.65
	182	Right superior parietal lobule; right precuneus	16	−74	52	3.56	3.43
	102	Right superior frontal gyrus	36	−6	60	3.42	3.30
	117	Left superior frontal gyrus	−24	−4	54	3.26	3.16
							
Direct & Averted > Downward	4851	Bilateral calcarine gyrus; left precuneus	−10	−66	6	5.51	5.08
	314	Right insula	36	26	2	5.18	4.82
	327	Right superior frontal gyrus	26	−4	54	4.11	3.91
	425	Right post/precentral gyrus; right Rolandic operculum	60	−4	22	4.00	3.82
	536	Left mid-cingulate cortex; left posterior-medial frontal cortex	−6	2	44	3.99	3.81
	542	Right postcentral gyrus; right superior/inferior parietal lobule	40	−40	64	3.97	3.79
	759	Left post/precentral gyrus; left inferior parietal lobule	−34	−30	56	3.76	3.61
	182	Right superior parietal lobule	−20	−52	60	3.60	3.47
	130	Right central sulcus	28	−20	52	3.57	3.44
	75	Left inferior parietal lobule	−36	−38	46	3.50	3.38
	92	Left post/precentral gyrus	−56	−8	16	3.44	3.32
	76	Left superior frontal gyrus	−26	−10	62	3.37	3.26
	122	Left IFG (pars opercularis, triangularis); left precentral gyrus	−44	10	28	3.36	3.25
	73	Left middle occipital gyrus	−42	−80	−2	3.29	3.18
	93	Right inferior temporal gyrus; right fusiform gyrus	52	−50	−16	3.28	3.17
	79	Right cerebellum (lobules VI; IV-V); cerebellar vermis	24	−54	−24	3.25	3.15

Conjunction null: (Direct < Averted & Downward) ∩ (3 channels > 2 channels)	153	Left lingual gyrus	−16	−56	2	3.85	3.69
	183	Left IFG (pars triangularis)	−42	24	−2	3.61	3.47
	83	Right insula	36	26	2	3.32	3.21
	66	Right calcarine gyrus	16	−56	4	3.14	3.05

**Table 2:** Results of the laterality index calculations (using the LI toolbox in SPM; Wilke and Schmithorst, 2006) for main effects of Gaze Direction and Auditory Clarity, and for the directional contrasts of 3 Channels>2 Channels and Direct>(Averted and Downward). All weighted means were significantly left lateralized (i.e. exceeding the conventional LI threshold of 0.2; Seghier et al., 2008).

Model	Contrast	Trimmed Mean	Min	Max	Weighted Mean
3×2 within-subjects ANOVA	Main Effect of Gaze Direction	0.4	0.18	0.65	0.62
	Main Effect of Auditory Clarity	0.44	0.29	0.62	0.5
	3 Channels > 2 Channels	0.25	0.03	0.52	0.42
	Direct > (Averted & Downward)	0.24	0.08	0.58	0.49

One-way within-subjects ANOVA	Direct > (Averted & Downward)	0.25	0.2	0.38	0.44

(2 Channels)					
One-way within-subjects ANOVA	Direct > (Averted & Downward)	0.05	−0.01	0.09	−0.09

(3 Channels)					

Additional activations for this effect of Auditory Clarity (and for the directional T-contrast of 3 Channels >2 Channels; see Table 1) included superior frontal cortex, a large cluster in bilateral calcarine gyrus and cuneus, bilateral sensorimotor cortex and regions of medial prefrontal cortex including the cingulate gyrus.

### 3.2 The left anterior temporal lobe is preferentially sensitive to socially salient gaze during speech perception

**Fig. 4:**
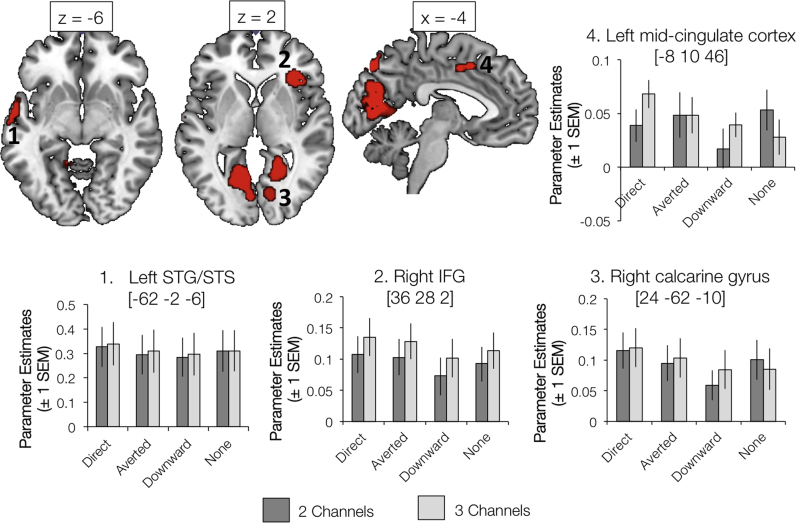
Significant clusters showing a main effect of Gaze Direction. Activations are shown at a voxel height threshold of p<0.005 and a corrected cluster extent threshold of p<0.001 (Slotnick et al., 2003). Plots show parameter estimates (±1 S.E.M.) taken from 4 mm-radius spherical regions-of-interest built around selected peak voxels (using the MarsBaR toolbox in SPM; Brett et al., 2002). Coordinates are given in Montreal Neurological Institute stereotactic space.

**Fig. 5:**
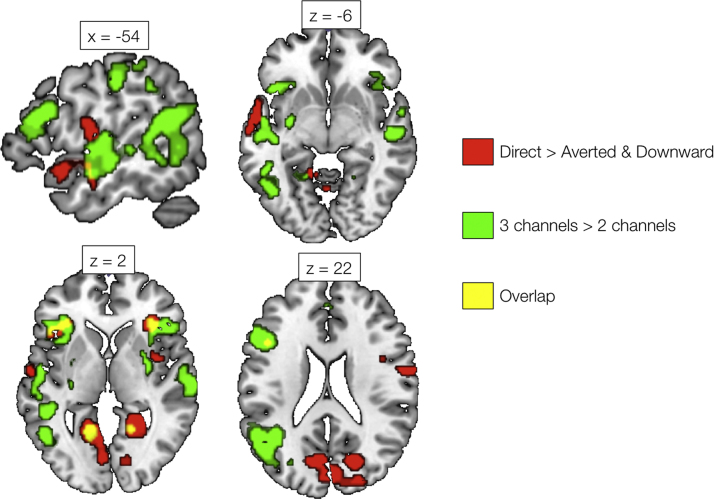
Significant clusters showing a preferential response to Direct Gaze (compared with Averted Gaze and Downward Gaze; red). Also shown are regions showing a significant effect in the contrast 3 Channels >2 Channels (Auditory Clarity factor; green). Yellow shading indicates regions of overlap. Activations are shown at a voxel height threshold of p<0.005 and a corrected cluster extent threshold of p<0.001 (Slotnick et al., 2003). Coordinates are given in Montreal Neurological Institute stereotactic space.

**Fig. 6:**
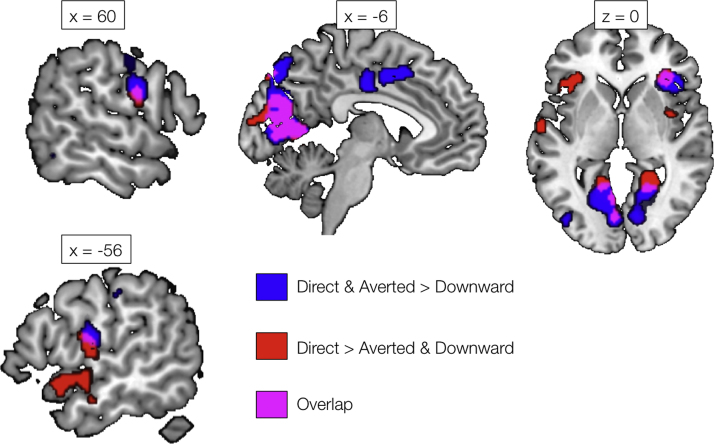
Significant clusters showing a preferential response to Direct and Averted Gaze (compared with Downward Gaze; blue). Also shown are regions showing a preferential response to Direct Gaze (compared with Averted Gaze and Downward Gaze; red). Magenta shading indicates regions of overlap. Activations are shown at a voxel height threshold of p<0.005 and a corrected cluster extent threshold of p<0.001 (Slotnick et al., 2003). Coordinates are given in Montreal Neurological Institute stereotactic space.

Fig. 4 illustrates regions of significant activation for the main effect of Gaze Direction (see also Table 1), including regions of visual, parietal and prefrontal cortex. The general trend was for greater responses to conditions where the sclera and pupils were visible; that is, the Direct and Averted Gaze conditions. Several regions were found to be more active in response to sentences in which the talker was looking directly at the camera (and, hence, the viewer) compared with averted and downward gaze (Fig. 5). Many of these regions overlapped with significant clusters in a contrast exploring shared preference for either direct or averted gaze (Table 1, Fig. 6), and included areas of inferior and superior frontal, visual, sensorimotor and parietal cortex. However, a region in the anterior temporal lobe (extending to temporal pole) showed a distinct preference for direct gaze and, like the preferential response to increased auditory clarity, showed a strong left lateralization (Table 2). In the 3×2 ANOVA model, all weighted LIs for the Main Effect of Gaze Direction, Main Effect of Auditory Clarity, 3 Channels >2 Channels and Direct >(Averted and Downward) contrasts were strongly left dominant in the superior temporal lobe (See Table 2). Preferential responses to averted gaze direction were mainly confined to regions of the right superior frontal and parietal cortices (Table 1).

There were no significant clusters showing an interaction of Auditory Clarity and Gaze Direction in the 3×2 ANOVA. However, analysis of temporal lobe lateralization within the T maps for the contrast Direct >(Averted and Downward) at the two different levels of Auditory Clarity showed that this effect was strongly left lateralized at 2 Channels (weighted mean=0.44) but showed no lateralization at 3 Channels (weighted mean=−0.09; see Table 2, Table 3). This suggests that when there were fewer available cues to the acoustic content of speech, participants’ responses to direct gaze were more strongly expressed in the left temporal lobe (presumably due to greater attention to the face to assist the speech comprehension task).

**Table 3:** Results of the contrast Direct>(Averted and Downward) at each level of Auditory Clarity (2 Channels, 3 Channels), as tested within one-way within-subject ANOVAs with the factor Gaze Direction (Averted, Direct, Downward). All results are reported at a voxel height threshold of p<0.005 (uncorrected), and a corrected cluster threshold of p<0.001 (Slotnick et al., 2003). Coordinates are given in Montreal Neurological Institute (MNI) stereotactic space. WM = white matter.

Auditory clarity	No of voxels	Region (s)	Peak coordinate			F/T	Z
			x	y	z		
2 Channels	378	Left pre/postcentral gyrus	−28	−16	48	5.19	4.43
	221	Right calcarine gyrus, right lingual gyrus	26	−60	12	4.66	4.07
	530	Left calcarine gyrus	−12	−62	6	4.26	3.79
	82	Right medial frontal WM	20	34	26	4.15	3.71
	222	Right pre/postcentral gyrus	36	−28	66	4.11	3.68
	79	Right IFG (pars triangularis), insula	38	32	2	4.11	3.68
	127	Left temporal pole, superior temporal gyrus, middle temporal gyrus	−54	14	−14	3.91	3.53
	192	L/R mid-cingulate cortex	−4	−2	38	3.54	3.24
	167	Left IFG (pars triangularis)	−46	24	12	3.45	3.17
	106	Left calcarine gyrus, middle occipital gyrus	−4	−98	10	3.31	3.06

3 Channels	230	Right cerebellum (Crus 1), right fusiform gyrus	40	−48	−36	4.95	4.26
	301	Right Heschl's gyrus, Rolandic operculum	54	−6	6	4.33	3.84
	116	Cerebellar vermis	0	−68	−26	3.79	3.44
	160	Right calcarine gyrus	18	−54	4	3.72	3.38
	96	Left cerebellum (Lobule IV-V, VI)	−18	−46	−20	3.67	3.34
	93	Left Rolandic operculum, postcentral gyrus	−56	−6	8	3.56	3.26
	77	Left superior temporal gyrus, temporal pole, middle temporal gyrus	−62	−2	−8	3.48	3.19
	96	Right cerebellum (Lobule VI, IV-V)	30	−54	−26	3.10	2.89

### 3.3 Bilateral anterior insula and inferior frontal cortex show common responses to increases in intelligibility and perceived communicative intent of audio-visual speech

Fig. 5 overlays the significant activations showing preferential responses to direct gaze and increased auditory clarity, where overlap can be seen in anterior temporal cortex, bilateral calcarine gyrus, and bilateral inferior frontal cortex and insula. A conjunction null of these two contrasts resulted in four significant clusters, with peaks in left inferior frontal gyrus, right insula, left lingual gyrus and right calcarine gyrus.

## 4 Discussion

The main aim of the study was to explore whether task context could influence the hemispheric lateralization of neural responses to the perception of the talker's eye gaze in the temporal lobe, specifically the STS. Indeed, we observed a main effect of talker gaze direction (specifically, a preferential response to direct gaze in the anterior temporal lobe) that was strongly left-lateralized and partially overlapping with a similar anterior temporal response to increases in auditory intelligibility. On closer investigation, we found that the preferential response to direct gaze in anterior temporal cortex was strongly left-lateralized at lower levels of auditory speech intelligibility, but showed no hemispheric dominance when auditory clarity was increased. This suggests flexibility in the spatial distribution of processes across homologous brain regions (i.e. gaze processing in the right and left anterior temporal lobes; Carlin et al., 2011) during spoken communication, where in this case the responses to speech and direct gaze were expressed most strongly in the same cerebral hemisphere when attention to both the face and the voice were maximally important for performance of the task. Thus, in line with Senju and Johnson's (2009) proposal,^2^ we present the evidence for dynamic, task-dependent responses to gaze at this relatively early stage in the speech comprehension process (i.e. the extraction of speech from a degraded auditory input). However, this conclusion should be tempered somewhat by the lack of significant interaction in temporal cortex between the gaze and intelligibility manipulations in our main ANOVA on the fMRI data.

Observing a response to gaze direction in the left anterior temporal lobe is not completely at odds with previous research, despite that fact that this has emphasized right-lateralized effects (Calder et al., 2002, Calder et al., 2006, Pelphrey et al., 2004, Carlin et al., 2011, Carlin and Calder, 2013). Carlin and colleagues found head-view-invariant responses to gaze direction in the right anterior STS, but using a region-of-interest analysis found that this response was also present in the homologous part of the left hemisphere. Kampe et al. (2003) found that the conjunction of activations in response to auditory and visual communicative signals revealed a cluster in the left temporal pole (and not the right). Our results fall in line with previous work suggesting that the anterior STS is the locus of extracting the social meaning of gaze (Carlin and Calder, 2013) – however, based on the work of Carlin and colleagues, we did not predict engagement of posterior sites because our talker maintained the same, front-facing head position throughout. Other authors have previously discussed the STS as a site for the perception of social and communicative signals in the voice (Redcay, 2008, Shultz et al., 2012), and the left-lateralization of gaze and speech responses identified in this study suggests a potential common processing pathway in this region for extracting meaning from another's actions (here, the movements of the mouth and the eyes during spoken communication). Notably, the BOLD signal in response to Downward gaze tended to be the smallest of the three gaze conditions (see plots of parameter estimates in Fig. 3, Fig. 4), and indeed lower than the Eyes Covered baseline. It could be argued that a downward glance was the least communicative visual stimulus presented - in the baseline stimuli, the participants may have maintained some expectation that a communicative gaze was being obscured by the masking rectangle (a reason why we did not include the Eyes Covered conditions in the Gaze Direction factor of our ANOVA analyses).

Direct gaze also engaged other structures previously implicated in studies employing gaze manipulations, including calcarine and fusiform gyri, and bilateral insula/IFG (Calder et al., 2002, Callejas et al., 2014, Ethofer et al., 2011, Pitskel et al., 2011). We did not observe ventromedial prefrontal cortex, as seen by Kampe et al. (2003) and Calder et al. (2002). Kuzmanovic et al. (2009) found greater activation of medial prefrontal cortex (mPFC) in response to direct gaze of increasing duration, and Bristow and colleagues (2007) found that regions of the “social brain” in mPFC and precuneus were engaged by attentional cueing gaze shifts that followed socially salient direct eye contact. The lack of medial prefrontal engagement in the current study may be an effect of the task demands, where our participants’ primary goal was to extract and comprehend the linguistic message and not to make an overt social judgement. Even in the absence of a social task, many previous studies have presented visual-only stimuli, and so the modulation of visual social cues was more prominent in those studies. Furthermore, the BKB sentences used in our study, which either refer to people in the 3rd person (e.g. “They’re pushing an old car”) or to scenes without human agents (e.g. “The rain came down”) did not provide a strong sense of social context.

Averted gaze can have strong social and informational salience. A glance to the side can signal that another individual is being addressed (e.g. Holler et al., 2015), or that the talker wishes the viewer to pay attention to a particular part of space (e.g. Bristow et al., 2007; Pelphrey et al., 2003; Ramsey et al., 2011). It can also potentially signal internal states in the talker (e.g. direct gaze in anger, averted gaze in sadness; Adams and Kleck, 2003). In the current experiment, preferential responses to averted gaze were found in right-dominant regions of the superior parietal lobe (including the intraparietal sulcus) and bilateral superior frontal gyri. This may correspond to engagement of the dorsal attentional network (DAN; Callejas et al., 2014; Corbetta et al., 2008), where averted gaze stimulates the expectation of an object or event at a lateralized location in space (Ramsey et al., 2011). In a simple sentence comprehension task, Staudte and colleagues (2014) observed that (averted) gaze direction aids comprehension through visual cueing (e.g. signaling the location of an object) and not by signaling the speaker's intentions. As the current study provided no explicit social context associated with averted gaze, our averted stimuli may similarly have been perceived as attentionally directing rather than strongly communicative (compared with direct gaze stimuli).

Our study revealed sensitivity to both the direction of gaze and the availability of additional auditory information in parts of the anterior insula bilaterally, extending into the inferior frontal gyrus. Similarly to effects in the temporal lobe, the involvement of the anterior insula and inferior frontal cortex in the perceptual processing of speech has tended to be left dominant (Davis & Johnsrude, 2003; Eisner et al., 2010; McGettigan et al., 2012a, b). Engagement of (bilateral) inferior frontal sites in the current experiment could partly reflect an overall stronger focus on the linguistic task in speech-processing regions in response to attentional capture by the eyes. The perception of direct eye contact is highly salient (Senju and Johnson, 2009) and communicatively important (Kampe et al., 2003) - the ventral attention network (VAN), implicating inferior frontal cortex, has been shown to be responsive to the occurrence of unexpected and important events (Corbetta et al., 2008). Inclusion of an attentional task demands, in future work, for example to attend to the mouth on some trials and the eyes on others, may allow us to tease apart cue-specific from domain-general aspects of attentional engagement with gaze in our stimuli. Such studies will be dependent on eye tracking data to verify adherence to the task (and to allow us to test predictions about eye contact vs. general gaze perception; see Footnote 1).

In recent years, an emerging literature has employed manipulations of gesture, gaze and body posture to investigate language comprehension in different communicative contexts. As in our study, there is evidence for varied distribution of activation depending on task and stimulus contexts. An enhanced response to direct gaze during spoken sentence comprehension was observed in right MTG (Holler et al., 2015), but only for conditions including speech and gesture (and not speech alone) – this was interpreted as evidence for the integration of meaning from multimodal inputs in the right temporal lobe. Straube et al. (2010) showed widespread enhancement of BOLD responses to stimuli in which the participant was directly addressed (i.e. speaker facing toward the viewer), which was larger in bilateral anterior temporal regions for sentences containing person-related (versus object-related) information. That these studies did not show similar left-dominant temporal responses to direct gaze, as seen in our study, may be due to a number of factors - the increased difficulty of our speech comprehension task potentially loaded more strongly on lower-level speech perception processes, and the use of face-only (rather than whole body) visual stimuli may have resulted in more focused attention on gaze direction. Further, it could be argued that previous studies using unmanipulated (i.e. clear) speech, as well as contrasts of semantic and pragmatic sentence properties, might measure relatively higher-order social and cognitive processes than the current study. However, it is also important to note here that while we have identified evidence that left-lateralization of responses to gaze direction became stronger when auditory intelligibility was reduced, thus suggesting an effect of speech processing on the response to gaze, we cannot make claims about the overall left-lateralization per se in relation to the speech communicative context. In order to support such a claim, we would have had to include some visual-only stimuli, and a non-speech baseline task – such modifications of the design should be implemented in future work.

Several recent studies have more closely examined social processing in language comprehension by considering the effects of participants’ subjective experience of the contextual manipulations. For example, Nagels et al. (2015) found that activations in the anterior cingulate cortex, fusiform gyrus, SMA, IFG and insula were positively correlated with subjective feelings of being addressed by the speaker (which was varied through manipulations to the talker's body posture and the use of co-speech gestures). In a study of gesture and written language comprehension, it was found that responses to participant-directed gestures (compared with non-communicative gestures) in regions of left STS were related to participants’ subjective ratings of communicative intent in the gesture stimuli – the same regions also showed greater responses to participant-directed sentences (compared with 3rd-person sentences; Redcay et al., 2016). Finally, a recent study that modulated the participant's belief about the presence of live and recorded interlocutors showed increased signal within mentalizing regions including mPFC and TPJ, and that the strength of the participant's subjective experience of the “live” speech was associated with increased responses across the mentalizing network (Rice and Redcay, 2016). Although it is accepted that direct gaze forms a salient social and communicative cue, the current study did not present participants with an explicit social context for the different gaze conditions used in the task. Future developments of this work could involve more direct manipulation of context (e.g. presenting the Averted Gaze condition as signaling speaker intention to another viewer; Holler et al., 2015) as well as manipulation of the sentence content to compare higher and lower levels of participant-directedness. Crucially, collecting subjective ratings of communicative intent would allow for stronger claims as to the social significance of gaze and sentence content, which could potentially be explored at a wider range of speech intelligibility levels than explored in the current experiment.

## 5 Conclusions

We report the first neuroimaging study to explore the interaction of talker gaze direction with the extraction of intelligible speech from degraded auditory inputs. We found that, when listening to degraded speech, the neural response to direct gaze in the STS that has previously been reported as predominately right-lateralized was lateralized to the left hemisphere, specifically at lower levels of auditory speech intelligibility. This finding supports Senju and Johnson's (2009) view of flexibility in the distribution of gaze-related activations around the cerebral cortex, depending on task demands, and with the argument that the human STS is optimized for the processing of social cues (Redcay, 2008). While the literature to date has associated speech more closely with linguistic computations, we argue that speech is fundamentally social, and that future research should aim to more closely examine how the context of spoken interactions both affords and affects social processing across the brain. More widely, our view aligns with an emerging movement in the social neuroscience literature arguing that, in order to truly understand the social brain, we should strive to investigate social processes in real time and within naturalistic interactions (a “second-person neuroscience”: e.g. Schilbach et al., 2014; McGettigan, 2015).

## Acknowledgements

We dedicate this paper to the memory of our colleague Andy Calder. This work was supported by a Wellcome Trust Senior Research Fellowship (WT090961MA) awarded to Sophie Scott.

## Footnotes

1 Age information was incorrectly recorded for one participant, who does not contribute to this mean. However, information from the participant database confirms that the participant's age was in the range 18–31 years old

2 Here, we refer only to Senju and Johnson's (2009) descriptions of task-dependent responses to gaze direction rather than to eye contact - due to a lack of usable eye tracking data, we cannot tease apart direct gaze perception from the perception eye contact in the current study.
