# Supplementary material for: You talkin’ to me? Communicative talker gaze activates left-lateralized superior temporal cortex during perception of degraded speech
Source: Neuropsychologia. 2017 Jun;100:51–63. doi: 10.1016/j.neuropsychologia.2017.04.013 (PMC5446325; doi:10.1016/j.neuropsychologia.2017.04.013)
Supplement: Supplementary file 1 — Supplementary material [file mmc1.docx]

**Supplemental Materials**

**Supplemental Results**

In order to explore pairwise relationships between the different gaze conditions and their interactions with auditory speech intelligibility, three within-subjects flexible factorial ANOVAs were run with the factors Gaze Direction and Auditory Clarity, for the following pairs of gaze conditions: (i) Direct vs. Downward, (ii) Averted vs. Downward and (iii) Direct vs. Averted. These revealed several commonalities with the findings of the 3 x 2 ANOVA reported in the main Results section of the manuscript. There were also novel insights: there were some areas of significant interaction between Gaze Direction and Auditory Clarity, though not in predicted regions (i.e. superior temporal cortex), while the comparisons of Direct gaze with the other two gaze conditions revealed a preferential response to direct eye contact in the left anterior temporal lobe in both cases. The results of these analyses are reported in Supplemental Tables 1-3 below.

**Supplemental Table 1** Results of a 2 x 2 ANOVA exploring the main effects of gaze direction and auditory clarity for the Direct and Downward conditions only. All results are reported at a voxel height threshold of p < .005 (uncorrected), and a corrected cluster threshold of p < .001 (Slotnick et al., 2003). Coordinates are given in Montreal Neurological Institute (MNI) stereotactic space.

| **Contrast** | **No of Voxels** | **Region(s)** | **Peak Coordinate** | | | **F/T** | **Z** |
| --- | --- | --- | --- | --- | --- | --- | --- |
|  |  |  | **x** | **y** | **z** |  |  |
| Main Effect of Gaze Direction (F-test) | 3356  235  159  83  297  117  181  109  83  68  79 | Right/left calcarine gyrus  Right IFG (pars triangularis)  Left STG/STS/temporal pole  Right inferior parietal lobule  Right post/precentral gyrus  Left precuneus  Left IFG (pars triangularis)  Left posterior-medial frontal lobe  Left postcentral gyrus  Left mid cingulate cortex  Left IFG (pars triangularis) | 24  36  -62  62  60  -6  -44  -8  -54  -6  -46 | -60  28  -2  -52  -4  -82  24  8  -6  -12  20 | 10  2  -8  44  20  46  -2  46  16  36  16 | 30.13  27.2  23.06  22.01  18.58  18.33  16.76  15.65  14.62  13.8  13.25 | 4.7  4.5  4.19  4.1  3.79  3.77  3.61  3.5  3.38  3.29  3.22 |
| Main Effect of Auditory Clarity (F-test) | 1670  392  394  344  1004  235  159  193  69  97  189  70 | Left middle occipital gyrus; left STS/STG, angular gyrus, superior parietal lobule  Right/left posterior-medial frontal cortex; left superior medial gyrus  Right STG / Heschl’s gyrus  Left IFG (pars triangularis)  Left middle/superior frontal gyrus; left pre/postcentral gyrus  Left/right precuneus; right superior parietal lobule  Left superior frontal gyrus  Left STG  Right cerebellum (crus I)  Left cuneus  Left inferior parietal lobule, supramarginal gyrus  Left lingual gyrus | -30  4  66  -48  -24  -8  -16  -58  38  -2  -56  -14 | -74  20  -18  18  14  -60  48  -18  -48  -78  -44  -58 | 32  50  2  22  60  54  36  8  -36  28  38  2 | 27.63  23.35  19.4  18.08  17.79  15.85  15.58  15.55  14.84  14.13  13.27  12.6 | 4.53  4.21  3.87  3.74  3.72  3.52  3.49  3.49  3.41  3.33  3.22  3.14 |
| Interaction of Gaze Direction and Auditory Clarity (F-test) | 85 | Left superior occipital gyrus; left calcarine gyrus | -18 | -86 | 8 | 11.99 | 3.06 |
| Direct Gaze > Downward Gaze (T-test) | 4618  87  300  215  555  474  196  134  79  70  123  110  92  183  68  170 | Right/left calcarine gyrus; left/right cuneus; left precuneus; left lingual gyrus  Right cerebellum (Crus I)  Right IFG (pars triangularis)  Left STG/STS/temporal pole  Right pre/post-central gyrus; right Rolandic operculum  Left IFG (pars triangularis); left precentral gyrus  Left posterior-medial frontal cortex  Left postcentral gyrus  Left superior frontal gyrus  Cerebellar vermis (lobule VI)  Left mid cingulate cortex  Right cerebellum (lobules IV-V, VI)  Right temporal thalamus  Left postcentral gyrus  Right middle frontal gyrus  Left pre/postcentral gyrus | 24  38  36  -62  60  -44  -8  -54  -26  0  -6  24  14  -36  30  -40 | -60  -48  28  -2  -4  24  8  -6  62  -62  -12  -54  -18  -30  -4  -6 | 10  -38  2  -8  20  -2  46  16  14  -24  36  -24  20  58  52  46 | 5.49  5.49  5.22  4.8  4.31  4.09  3.96  3.82  3.78  3.74  3.72  3.56  3.41  3.34  3.28  3.24 | 4.84  4.84  4.65  4.34  3.96  3.79  3.68  3.57  3.53  3.5  3.48  3.35  3.22  3.16  3.11  3.07 |
| Downward Gaze > Direct Gaze (T-test) | 104  68 | Right inferior parietal lobule  Left medial temporal white matter | 62  -26 | -52  -40 | 44  22 | 4.69  3.08 | 4.26  2.94 |
| 3 Channels > 2 Channels (T-test) | 4287  3022  759  96  245  124  181  133  140  83  108  92 | Left middle occipital gyrus; left STS/STG, superior parietal lobule, angular gyrus, inferior parietal lobule; left precuneus.  Right/left posterior-medial frontal cortex; left IFG (pars triangularis); left middle/superior frontal gyrus; left precentral gyrus.  Right superior temporal gyrus / Heschl’s gyrus  Left posterior insula  Left superior frontal gyrus  Right cerebellum (Crus 1)  Left cuneus  Left lingual gyrus  Left cerebellum (lobule VI); left lingual gyrus  Left superior medial gyrus  Right insula; right IFG (pars triangularis, pars orbitalis)  Left inferior parietal lobule; left postcentral gyrus | -30  4  66  -32  -16  38  -2  -14  -18  -4  38  -56 | -74  20  -18  -6  48  -48  -78  -58  -56  42  22  -20 | 32  50  2  -8  36  -36  28  2  -16  52  -8  38 | 5.26  4.83  4.4  4.04  3.95  3.85  3.76  3.55  3.41  3.41  3.19  3.06 | 4.68  4.36  4.04  3.74  3.67  3.59  3.51  3.34  3.22  3.22  3.03  2.91 |
| 2 Channels > 3 Channels (T-test) | 81  92 | Right medial temporal white matter / CSF  Left medial temporal white matter / CSF | 12  -20 | -36  -34 | 24  22 | 3.45  3.21 | 3.25  3.05 |

**Supplemental Table 2** Results of a 2 x 2 ANOVA exploring the main effects of gaze direction and auditory clarity for the Averted and Downward conditions only. All results are reported at a voxel height threshold of p < .005 (uncorrected), and a corrected cluster threshold of p < .001 (Slotnick et al., 2003). Coordinates are given in Montreal Neurological Institute (MNI) stereotactic space.

| **Contrast** | **No of Voxels** | **Region(s)** | **Peak Coordinate** | | | **F/T** | **Z** |
| --- | --- | --- | --- | --- | --- | --- | --- |
|  |  |  | **x** | **y** | **z** |  |  |
| Main Effect of Gaze Direction (F-test) | 301  242  1184  85  252  115  86 | Left calcarine gyrus  Right superior/middle frontal gyrus  Right insula  Right postcentral gyrus; right superior/inferior parietal lobule; right precuneus  Right calcarine gyrus; right lingual gyrus  Left superior parietal lobule; left precuneus  Left precentral gyrus; left superior frontal gyrus | -10  26  38  38  18  -16  -36 | -68  -2  -46  26  -70  -52  -10 | 8  54  64  2  12  60  54 | 25.1  22.17  18.91  17.24  16.84  16.73  14.93 | 4.35  4.11  3.82  3.66  3.62  3.61  3.42 |
| Main Effect of Auditory Clarity (F-test) | 667  838  364  153  174  601  78  196  98  160  85 | Left pre/postcentral gyrus; left superior frontal gyrus; left paracentral lobule  Left inferior/middle temporal gyrus  Left/right mid cingulate cortex  Left/right superior medial gyrus  Left medial temporal white matter / CSF  Left IFG (pars triangularis); left precentral gyrus  Right middle/inferior temporal gyrus  Right pre/postcentral gyrus  Left calcarine gyrus  Left inferior parietal lobule; left postcentral gyrus  Left STG/STS | -28  -46  -4  -4  -30  -50  48  56  -12  -46  -46 | -18  -58  4  54  -48  28  -54  -12  -62  -28  -22 | 48  -10  36  36  6  6  -2  44  4  42  -4 | 19.88  17.92  16.18  16.03  15.52  14.57  14.18  13.72  13.12  12.07  11.79 | 3.91  3.73  3.55  3.54  3.48  3.38  3.33  3.28  3.21  3.07  3.04 |
| Interaction of Gaze Direction and Auditory Clarity (F-test) | 79  113  69  113 | Left superior occipital gyrus; left calcarine gyrus  Left superior occipital gyrus  Left middle occipital gyrus  Right cuneus; right calcarine gyrus | -16  -18  -38  16 | -86  -84  -86  -86 | 8  44  16  40 | 15.71  14.36  14.14  12.5 | 3.5  3.35  3.33  3.13 |
| Averted Gaze > Downward Gaze (T-test) | 2468  365  129  419  256  249  87  79 | Left calcarine gyrus; right postcentral gyrus; right superior/inferior parietal lobule; right precuneus; right postcentral gyrus  Right middle/superior frontal gyrus  Right insula  Right calcarine gyrus; right lingual gyrus  Left superior parietal lobule; left precuneus  Left pre/postcentral gyrus; left superior frontal gyrus  Right postcentral gyrus  Left inferior parietal lobule | -10  26  38  18  -16  -36  26  -50 | -68  -2  26  -70  -52  -10  -20  -24 | 8  54  2  12  60  54  52  40 | 5.01  4.71  4.15  4.1  4.09  3.86  3.64  3.35 | 4.5  4.27  3.84  3.8  3.79  3.6  3.41  3.17 |
| Downward Gaze > Averted Gaze (T-test) | 81  70 | Left medial frontal white matter  Left medial frontal white matter | -22  -14 | -16  12 | 34  24 | 4.31  3.41 | 3.96  3.22 |
| 3 Channels > 2 Channels (T-test) | 1194  1584  720  307  206  953  195  93  449  256  417  212  116  159  141  84 | Left pre/postcentral gyrus; left superior frontal gyrus; left paracentral lobule  Left inferior/middle temporal gyrus  Left/right mid cingulate cortex; left posterior-medial frontal cortex  Left/right superior medial gyrus; left superior frontal gyrus  Right IFG (pars triangularis, pars orbitalis); right insula  Left IFG (pars triangularis); left precentral gyrus  Right middle/inferior temporal gyrus  Right STG  Right pre/postcentral gyrus; right middle frontal gyrus  Left/right calcarine gyrus  Left inferior parietal lobule; left postcentral gyrus  Left STG/STS  Right precentral gyrus; right IFG  Left superior/inferior parietal lobule  Right superior parietal lobule  Right STS | -28  -46  -4  -4  42  -50  48  50  56  -12  -46  -46  48  -30  28  44 | -18  -58  4  54  32  28  -54  -16  -12  -62  -28  -22  6  -64  -60  -48 | 48  -10  36  36  6  6  -2  -6  44  4  42  -4  32  46  52  20 | 4.46  4.23  4.02  4  3.88  3.82  3.77  3.73  3.7  3.62  3.47  3.43  3.41  3.35  3.27  3.01 | 4.08  3.9  3.73  3.72  3.62  3.56  3.52  3.49  3.47  3.4  3.28  3.24  3.22  3.17  3.1  2.87 |
| 2 Channels > 3 Channels (T-test) | 284 | Left hemisphere white matter / CSF | -30 | -48 | 6 | 3.94 | 3.86 |

**Supplemental Table 3** Results of a 2 x 2 ANOVA exploring the main effects of gaze direction and auditory clarity for the Direct and Averted conditions only. All results are reported at a voxel height threshold of p < .005 (uncorrected), and a corrected cluster threshold of p < .001 (Slotnick et al., 2003). Coordinates are given in Montreal Neurological Institute (MNI) stereotactic space.

| **Contrast** | **No of Voxels** | **Region(s)** | **Peak Coordinate** | | | **F/T** | **Z** |
| --- | --- | --- | --- | --- | --- | --- | --- |
|  |  |  | **x** | **y** | **z** |  |  |
| Main Effect of Gaze Direction (F-test) | 102  139  115  159  87 | Right inferior parietal lobule  Right cerebellum (lobule IV-V); right fusiform gyrus; right parahippocampal gyrus  Left lingual gyrus; left cerebellum (lobule IV-V)  Right lingual gyrus  Left middle occipital gyrus | 64  30  20  -14  -16 | -48  -40  -48  -50  -92 | 42  -26  2  -10  14 | 27.44  25.33  21.14  19.43  14.11 | 4.52  4.36  4.03  3.87  3.32 |
| Main Effect of Auditory Clarity (F-test) | 494  119  614  182  149  82  250  158  74  756  246  69  75  97  83  68 | Right IFG; right insula  Left putamen  Left inferior parietal lobule; left postcentral gyrus  Left/right mid cingulate cortex  Left superior frontal gyrus  Right STG/STS  Left STS  Left inferior parietal lobule; left STS  Right putamen  Left precentral gyrus; left IFG (pars triangularis); left insula  Left medial white matter  Left superior frontal gyrus  Right superior medial gyrus; right posterior-medial frontal cortex  Left middle occipital gyrus  Right IFG (pars opercularis)  Left superior medial gyrus | 46  -32  -32  2  -22  52  -54  -38  30  -48  -16  -10  6  -30  44  -4 | 28  -8  -36  2  -6  -16  -18  -50  -20  6  -30  52  26  -72  12  42 | 6  -6  42  32  52  -4  -10  18  2  36  22  36  52  30  26  50 | 23.26  20.5  20.43  20.42  19.86  17.51  15.91  15.78  15.71  15.56  15.32  13.78  12.64  12.21  11.89  11.64 | 4.2  3.97  3.96  3.96  3.91  3.69  3.52  3.51  3.5  3.49  3.46  3.29  3.15  3.09  3.05  3.02 |
| Interaction of Gaze Direction and Auditory Clarity (F-test) | 70 | Right cerebellum (Crus 1) | 44 | -48 | -32 | 16.86 | 3.62 |
| Averted Gaze > Direct Gaze (T-test) | 130  78 | Right inferior parietal lobule  Left inferior parietal lobule | 64  -66 | -48  -24 | 42  38 | 5.24  4.36 | 4.66  4 |
| Direct Gaze > Averted Gaze (T-test) | 222  171  213  109  155  168  91 | Right cerebellum (Crus 1; lobules IV-V, VI); right fusiform gyrus  Right lingual gyrus  Left lingual gyrus; left cerebellum (lobules IV-V)  Right insula  Left temporal pole; left STG/STS  Left middle occipital gyrus; left calcarine gyrus  Left calcarine gyrus | 30  20  -14  44  -56  -16  -26 | -40  -48  -50  4  12  -92  -48 | -26  2  -10  6  -12  14  6 | 5.03  4.6  4.41  4.3  3.76  3.76  3.6 | 4.51  4.19  4.04  3.95  3.52  3.51  3.38 |
| 3 Channels > 2 Channels (T-test) | 684  177  1081  306  272  385  360  470  109  1270  74  127  132  125  102  169  128  102  140  155  185  140 | Right IFG (pars triangularis); right insula  Left putamen  Left inferior parietal lobule  Left mid cingulate cortex; left superior medial gyrus; left superior frontal gyrus  Left superior frontal gyrus; left precentral gyrus  Right STG/STS; right Rolandic operculum  Left STS  Left STS; left middle occipital gyrus  Right putamen  Left pre/postcentral gyrus; left IFG (pars triangularis); left insula  Right precuenus  Left superior frontal gyrus  Left lingual gyrus  Right/left precuneus  Left superior medial gyrus  Right superior medial gyrus; right posterior-medial frontal cortex  Right superior/middle frontal gyrus  Right postcentral gyrus  Right precentral gyrus  Left middle/inferior temporal gyrus; left middle/inferior occipital gyrus  Right IFG (pars opercularis)  Left superior medial gyrus | 46  -32  -32  2  -22  52  -54  -38  30  -48  18  -10  -14  4  0  6  12  42  52  -50  44  -4 | 28  -8  -36  2  -6  -16  -18  -50  -20  6  -40  52  -54  -52  44  26  54  -22  -8  -60  12  42 | 6  -6  42  32  52  -4  -10  18  2  36  46  36  2  62  30  52  36  36  44  2  26  50 | 4.82  4.53  4.52  4.52  4.46  4.18  3.99  3.97  3.96  3.94  3.83  3.71  3.7  3.61  3.59  3.56  3.55  3.54  3.53  3.5  3.45  3.41 | 4.36  4.13  4.13  4.12  4.08  3.86  3.7  3.69  3.68  3.67  3.57  3.48  3.47  3.39  3.37  3.34  3.34  3.33  3.32  3.3  3.25  3.22 |
| 2 Channels > 3 Channels (T-test) | 384  149 | Left medial temporal white matter / CSF  Right medial temporal white matter / CSF | -16  18 | -30  -32 | 22  22 | 3.91  3.32 | 3.64  3.14 |
